# CD19 CAR T cell therapy in systemic sclerosis: A case study that reaffirms the promise of cell therapy to treat autoimmune disease

**DOI:** 10.1016/j.omta.2026.201684

**Published:** 2026-02-17

**Authors:** James L. Riley

**Affiliations:** 1 Department of Microbiology, Center for Cellular Immunotherapies, Colton Center of Excellence for Cell Therapy in Autoimmunity, University of Pennsylvania, Philadelphia, PA 19104, USA

## Main text

Systemic sclerosis (SSc) remains one of the most challenging autoimmune diseases to treat, characterized by immune dysregulation, progressive fibrosis, vasculopathy, and high disease-related mortality. While conventional immunosuppressive and biologic therapies can slow disease progression in some patients, durable, drug-free remissions are rare, particularly in diffuse cutaneous SSc (dcSSc).^1^ In this issue of *Molecular Therapy Advances*, Nuñez and colleagues report a detailed case study of the first patient treated with resecabtagene autoleucel (rese-cel), a fully human CD19-directed chimeric antigen receptor (CAR) T cell therapy, within the RESET-SSc phase 1/2 clinical trial (NCT06328777).^2^ The authors describe not only encouraging clinical improvement but also an unusually deep and well-characterized depletion of B cells across peripheral blood and secondary lymphoid tissue. Beyond documenting feasibility and safety, this report provides important mechanistic insights into how CAR T cell-mediated B cell ablation may reset pathogenic immunity in SSc. As a very promising albeit single-patient analysis, the findings raise critical questions about durability, generalizability, and the relative contribution of lymphodepleting conditioning for the treatment of SSc with CAR T cells.

SSc is a prototypic systemic autoimmune disease in which aberrant crosstalk between immune cells, fibroblasts, and endothelial cells drives fibrosis and vascular damage. Among immune compartments, B cells have long been implicated in SSc pathogenesis. Disease-specific autoantibodies, including anti-RNA polymerase III, anti-topoisomerase I, and anti-centromere antibodies, are strongly associated with disease subsets and clinical outcomes.^3^ Beyond autoantibody production, B cells contribute through antigen presentation, cytokine secretion, and regulation of T cell responses. These observations have motivated therapeutic strategies aimed at B cell depletion. Rituximab, a CD20-targeting monoclonal antibody, has shown signals of efficacy in SSc, particularly for interstitial lung disease. However, randomized controlled trials have yielded mixed results, and responses are often partial and transient.^4^ One key limitation is incomplete depletion of B cells in secondary lymphoid tissues by rituximab, allowing autoreactive clones to persist and re-expand.^1^ In contrast, CAR T cell therapies targeting CD19 have the potential to eliminate B cells more comprehensively, including within lymphoid organs, raising the possibility of deeper immune “rebooting.”^5^

Early compassionate-use reports and small case series of CD19 CAR T cells in SSc and other autoimmune diseases have generated considerable excitement, demonstrating drug-free remissions in otherwise refractory patients. Nevertheless, most published experiences to date have limited correlative studies and treatment outside formal clinical trials. The current report by Nuñez et al., therefore, represents an important transition point: a mechanistically rich case study embedded within a prospective phase 1/2 trial framework.

The authors describe a 66-year-old woman with dcSSc, progressive skin disease, interstitial lung involvement, inflammatory arthritis, and anti-RNA polymerase III antibodies, refractory to multiple immunomodulatory therapies. Following standard lymphodepletion with fludarabine and cyclophosphamide, the patient received a single infusion of rese-cel at 1 × 10ˆ6 CAR T cells/kg. Safety outcomes were consistent with expectations from prior autoimmune CAR T experiences: a brief episode of grade 2 cytokine release syndrome (CRS), no immune effector cell-associated neurotoxicity syndrome, and transient grade 4 neutropenia that resolved rapidly.

Clinically, the patient demonstrated meaningful improvement in several areas during the nine months of followup, including a reduction in modified Rodnan skin score, improvements in pulmonary function and patient-reported outcomes, and nailfold capillaroscopy findings suggestive of vascular stabilization. Importantly, all immunosuppressive medications were discontinued at the time of CAR T infusion, underscoring the drug-free nature of the observed response. One of the most compelling aspects of this report is the depth and granularity of the immunologic analyses. Peripheral B cells became undetectable by day 13 post-infusion and remained absent for several weeks. Strikingly, paired inguinal lymph node biopsies demonstrated near-complete elimination of CD19+ cells within secondary lymphoid tissue, confirmed both by immunohistochemistry and spatial transcriptomics. This degree of tissue-level B cell depletion directly addresses a major limitation of antibody-based B cell therapies and provides strong support for the conceptual advantage of CAR T cells in autoimmune disease.

The authors further document a pronounced increase in serum B cell-activating factor (BAFF), reaching levels substantially higher than those reported following rituximab treatment. This marked BAFF elevation likely reflects a homeostatic response to profound B cell loss and may serve as a useful pharmacodynamic biomarker of deep tissue depletion. As B cells repopulated beginning around 8 weeks post-infusion, they exhibited a predominantly transitional naive phenotype, consistent with *de novo* reconstitution from bone marrow progenitors rather than expansion of residual memory clones. Equally notable is the serologic dissociation observed after therapy. Anti-RNA polymerase III antibodies declined to undetectable levels, whereas vaccine-induced antibodies were preserved. This pattern supports the emerging notion that pathogenic autoantibodies in autoimmune disease are maintained by CD19+ B cells or short-lived plasma cells, while long-lived protective humoral immunity resides in CD19− plasma cell compartments spared by CD19-directed CAR T therapy.

The study also provides informative data on CAR T cell expansion, phenotype, and cytokine dynamics. Although the infused product was predominantly CD4+ and enriched for central memory cells, circulating CAR T cells at peak expansion were largely CD8+ effector memory cells, suggesting differential expansion or trafficking patterns *in vivo*. Serum interferon-γ peaked early, preceding maximal CAR T cell detection in peripheral blood, consistent with rapid target engagement in tissues. Subsequent increases in interleukin-6 and other inflammatory cytokines coincided with CRS but remained substantially lower than levels typically observed in oncology settings, reinforcing the manageable safety profile seen in autoimmune CAR T applications.

This case study strengthens the rationale for CD19 CAR T cell therapy as a potentially transformative approach for severe, refractory SSc. By demonstrating deep B cell depletion in both blood and tissue, selective elimination of disease-associated autoantibodies, and coordinated clinical improvement, the report moves the field beyond anecdotal efficacy toward mechanistic plausibility. The RESET-SSc trial framework further enhances confidence that these observations will be systematically evaluated in additional patients. At the same time, several important caveats warrant emphasis. First, as a single-patient analysis, the findings cannot yet define efficacy, durability, or risk-benefit balance across the heterogeneous SSc population. Second, the contribution of lymphodepleting chemotherapy to early clinical and serologic changes cannot be fully disentangled, particularly in the short term. Third, the long-term consequences of profound, but transient, B cell ablation—including infection risk, hypogammaglobulinemia, and disease relapse after immune reconstitution—remain to be defined.

Looking ahead, key questions include whether specific autoantibody profiles or disease stages predict response, whether repeated or lower-dose CAR T strategies might optimize safety, and how CAR T cell therapy compares with or complements autologous hematopoietic stem cell transplantation. More broadly, this work exemplifies how gene and cell therapies originally developed for oncology can be repurposed to interrogate—and potentially reset—pathogenic immune circuits in autoimmune disease. If replicated in larger cohorts, the findings reported by Nuñez et al. may mark a conceptual shift in the treatment of SSc: from chronic suppression toward targeted, time-limited immune reprogramming.

## Declaration of interests

The author is a scientific co-founder of, holds equity in, and receives research funding from BlueWhale Bio and is eligible to receive milestone-based payments from Kite Pharma, a Gilead company.
